# The socioemotional challenges and consequences for caregivers of Aboriginal and Torres Strait Islander children with otitis media: A qualitative study

**DOI:** 10.1111/hex.13476

**Published:** 2022-03-16

**Authors:** Letitia Campbell, Jennifer Reath, Wendy Hu, Hasantha Gunasekera, Deborah Askew, Chelsea Watego, Kelvin Kong, Robyn Walsh, Kerrie Doyle, Amanda Leach, Claudette Tyson, Penelope Abbott

**Affiliations:** ^1^ Kalwun Development Corporation Gold Coast Australia; ^2^ Department of General Practice School of Medicine Western Sydney University Penrith Australia; ^3^ Faculty of Medicine and Health University of Sydney New South Wales Australia; ^4^ Primary Care Clinical Unit, School of Clinical Medicine The University of Queensland Brisbane Australia; ^5^ School of Public Health and Social Work, Faculty of Health Queensland University of Technology Brisbane Australia; ^6^ School of Medicine and Public Health College of Health, Medicine and Wellbeing, University of Newcastle Newcastle Australia; ^7^ Child Health Division Menzies School of Health Research Casuarina Australia; ^8^ Southern Queensland Centre of Excellence in Aboriginal and Torres Strait Islander Primary Health Care (Inala Indigenous Health Service), Metro South Health Brisbane Australia

**Keywords:** Aboriginal and Torres Strait Islander Health, child health, delivery of healthcare, hearing loss, otitis media

## Abstract

**Introduction:**

Living with ear disease can have extensive impacts on physical, emotional and social well‐being. This study explored otitis media (OM) and its management from the perspective of caregivers of Aboriginal and Torres Strait Islander children.

**Methods:**

Semi‐structured interviews were conducted from 2015 to 2020 with caregivers of Aboriginal and Torres Strait Islander children with OM. Thematic analysis of transcripts was undertaken using a constructivist grounded theory approach through the leadership and the cultural lens of an Aboriginal community‐based researcher.

**Results:**

Caregivers described OM as having profound impacts on their child's physical, developmental, and emotional well‐being, with long waits for specialist treatment contributing to extra strain on families. Children's well‐being suffered when OM was mistaken for poor behaviour and children were punished, with caregivers subsequently experiencing strong feelings of guilt. Concerns were conveyed about the social implications of having a sick child. The variable nature of OM symptoms meant that caregivers had to monitor closely for sequelae and advocate for appropriate treatment. Success in navigating the diagnosis and treatment of OM can be strongly impacted by the relationship between caregivers and health professionals and the perceived access to respectful, collaborative and informative healthcare.

**Conclusion:**

OM may have substantial social and emotional consequences for children and their caregivers. A holistic understanding of the way in which OM impacts multiple facets of health and well‐being, as well as recognition of challenges in accessing proper care and treatment, will aid families managing OM and its sequelae.

**Patient or Public Contribution:**

Governing boards, managers, staff and community members from five Australian Aboriginal Medical Services were involved in the approval, management and conduct of this study and the wider clinical trials. The caregivers of Aboriginal and Torres Strait Islander patients at these services informed the interview study and guided its purpose.

## 1 INTRODUCTION

Living with chronic middle ear disease, and managing its sequelae, is a common reality for many Aboriginal and Torres Strait Islander children and their families.^1^, ^2^, ^3^ Middle ear disease (otitis media [OM]) encompasses all inflammation or infection of the middle ear including acute infection (AOM), OM with effusion (OME) and acute or chronic discharge of pus through a hole in the tympanic membrane (including chronic suppurative OM [CSOM]).^3^ OM is regarded as a complex disease, most commonly experienced in childhood, with the potential to result in short‐ or long‐term consequences for hearing, language development, school performance and behaviour.^1^, ^4^, ^5^

The physical impacts of the disease, such as pain, are important to consider in improving outcomes for children with OM, but so too is the impact of OM on the social and emotional well‐being of children, as well as their caregivers and their ability to manage their child's OM.^1^, ^6^, ^7^ OM is often a silent disease, and symptoms can be subtle and detected only by caregivers understanding the disease and closely monitoring hearing‐related behaviour.^1^, ^5^ Studies of the impact of OM typically focus on direct costs in high‐income countries or communities,^8^ despite OM rates and the potential social costs being disproportionately high in First Nations populations.^2^, ^6^, ^9^, ^10^, ^11^ Qualitative explorations of OM experiences for the Aboriginal and Torres Strait Islander populations in Australia have been very limited, so the impact of OM on these families is not well understood.^6^ In this study, an Aboriginal researcher led the exploration of the OM‐related experiences of caregivers of Aboriginal and Torres Strait Islander children and the impact of OM and related healthcare on their health and well‐being.

## 2 CONTEXT

*Social/political/historical context*: Government policies of segregation, assimilation and child removal of Aboriginal and Torres Strait Islander peoples, the First Nations people of Australia, have had far‐reaching and ongoing negative consequences across generations in terms of socioeconomic status, health, education, employment and contact with the criminal justice system.^12^, ^13^ Systemic racism, lack of culturally appropriate care and decreased trust of healthcare institutions create ongoing barriers to accessing the necessary resources for achieving better health.^12^ The health disparities include a high prevalence of OM and conductive hearing loss, which in turn drives the vicious cycle of socioeconomic disadvantage in education, employment and well‐being for Aboriginal and Torres Strait Islander peoples.^3^ There is a need for OM research that engages with Aboriginal and Torres Strait Islander children and families to identify and address the gaps and tailor healthcare and information.^14^

*Research context*: Health research has reflected the same institutional and structural biases evidenced in the health system, relegating Aboriginal and Torres Strait Islander peoples to objects of study, with nonindigenous persons controlling the narrative of investigation and publication.^14^ Research conceived and led by First Nations peoples is essential to decolonize the western processes of racialized knowledge production of Indigenous deficit.^15^ In accordance with an Indigenist Standpoint,^16^ this study seeks to privilege the lived experience and knowledge of caregivers of Aboriginal and Torres Strait Islander children while uncovering the systemic failings that perpetuate notions of Indigenous deficit, parenting or otherwise. This study has been conducted with Indigenous expertize in ear‐health, race and indigenous methodologies in accordance with the principle of political integrity,^16^ which asserts that Aboriginal and Torres Strait Islanders must be at the heart of the research process and decision‐making.

Following the principles of ethical research with Aboriginal and Torres Strait Islander people,^17^ this study was conceived as a result of conversations between community members and an Aboriginal research officer during screening for two ongoing randomized controlled trials (RCTs)—the WATCH and INFLATE trials.^18^ The WATCH trial compares watchful waiting to immediate antibiotics for the treatment of AOM without perforation in Aboriginal and Torres Strait Islander children living in urban areas. The INFLATE trial investigates a nasal balloon autoinflation to treat OME in Aboriginal and Torres Strait Islander children. WATCH and INFLATE were run concurrently and involved the participation of Australian Aboriginal (and Torres Strait Islander) Medical Services (AMSs). AMSs are multidisciplinary primary health services that are typically board‐governed and community‐controlled to deliver culturally appropriate care for their local Aboriginal and Torres Strait Islander community. Participants were drawn from five AMSs in Brisbane, Gold Coast, Townsville, Sydney and Canberra. Research officers, usually Aboriginal and Torres Strait Islander people from the local community, were employed by the AMS to coordinate trial processes, including recruitment, follow‐up and data collection.

## 3 METHODS

### 3.1 Ethical consultation and approval

The research involved Indigenous community consultation and involvement in accordance with the National Guidelines for Ethical Conduct in Aboriginal and Torres Strait Islander Health Research.^19^ Senior Indigenous health researchers formed part of the broader investigative team, and participating AMSs were encouraged and supported to have Aboriginal and Torres Strait Islander advisory groups to review progress and provide guidance on the trials generally and specifically to their community. In Australia, ethical standards require an identified Aboriginal and Torres Strait Islander person(s) to be present on ethics committees approving any work conducted with this population.^20^ Approval was sought and received from AMS Boards or designated research committees and from human research ethics committees including (a) the Aboriginal Health and Medical Research Council Ethics Committee (938/13), (b) Western Sydney University (H10369), (c) Department of Health and Menzies School of Health (13/2074), (d) Metro South Human Research Ethics Committee (HREC/13/QPAH/366) and (e) The University of Queensland (2013001093).

### 3.2 Research team

The research team included Aboriginal and nonindigenous researchers involved in the WATCH and INFLATE trials. The project was conceived with the community and led by L. C., an emerging Aboriginal researcher and WATCH/INFLATE research officer. L. C. fulfilled a complex role as both insider and outsider to the research. Insider–outsider status is considered to be a continuum whereby researchers reflect on their relationship with participants considering the influence of prior knowledge, power and shared experiences on the collection and interpretation of data.^21^ L. C. was an insider, first, as an Aboriginal person with shared history with some of the interview participants, and a member of one of the communities participating in the research, second, in terms of considerable acquired knowledge of OM diagnosis, treatment and presentation and third, as a staff member of the AMS (>4 years). However, L. C. was also an outsider, with no personal or family history of OM, was funded to conduct the research by a nonindigenous institution and had a staff role in only one community involved in this study. Other authors included another AMS research officer, and researchers with expertize in qualitative research, clinical trials and clinician researchers, four of whom are Aboriginal researchers.

### 3.3 Participants

Participants included parents or carers who consented for their child to be screened for the WATCH and INFLATE trials and will hereafter be referred to as ‘caregivers’ to allow inclusiveness of the varied care arrangements of these families. Screening for the trials involved otoscopy and tympanometry testing of the child typically by a research officer. There were two groups of participants. One group included caregivers of children eligible to participate in the WATCH or INFLATE trials including caregivers who declined RCT participation but agreed to be interviewed. The second group included caregivers of children ineligible for the RCT because of complex ear disease. The first group of participants was recruited between 2015 and 2020 as part of the RCT's process evaluation, and the second group was recruited by L. C. in 2020 using purposive sampling to include caregivers with a broader range of OM experiences and diverse family situations. All participants provided informed consent before any data collection.

### 3.4 Data collection

Semi‐structured interviews were conducted over the phone or in person in a private room in the participants' AMS. Expert knowledge and a review of literature informed the development of the interview schedule (see Table 1). Four team members conducted the interviews with RCT‐eligible caregivers, none having any other direct involvement with these families. L. C. conducted eight interviews with caregivers of children with complex ear disease who were ineligible for the RCT. L. C. had prior involvement with the families attending her AMS as a staff member and as a member of the local community. Four researchers had qualitative interview experience including L. C.; one was a novice. Two interviewers were Aboriginal. The AMS research officers were present in some face‐to‐face interviews when preferred by participants. A yarning approach was adopted by interviewers with the experience to do so.^22^, ^23^

**Table 1:** Interview question guidelines

Question	Subquestion/prompts
*Child's experience*	
Has your child(ren) ever had an AOM/ear ache before?	How many ear infections/aches have they had?
	How old were they when they had their first one?
What was the worst ear infection they ever had? Can you tell me about that?	And what about one that wasn't that bad, can you tell me how that was different?
What effects/impacts have these ear problems had on the child/you/the family?	Prompts if needed: Time off work, hearing, behaviour/playfulness, school performance, financial costs
*Symptoms*	
How did you know they had an ear infection?	What are the tell‐tale signs that your child has an ear infection? Is it the same every time?
*Treatment response*	
How do you decide whether to go to a doctor or not?	
Thinking of a time you did go to the doctor, what happened during the consultation?	Were you given a choice of treatment?
	Did you get antibiotics? Which one? Did it work? 1 Dose or multiple?
	How did you feel about that treatment? Is that what you prefer? Why?
So the other times they've had an ear infection, did you do the same thing?	
Do you have any home remedies you use to treat the ear ache/infection?	
When a child has a middle ear infection, do you have any views on what treatment should be given?	
*Wider personal experience*	
Have you or other family members had any other experience of middle ear infections before?	What happened? How was it treated?
	Do you feel it has affected you as an adult?
	What about hearing problems in the family?
	Do you think they should be given antibiotics? What makes you think this way?
What do you think about ear disease in your community?	Do you think ear disease is a normal part of childhood?

All caregivers completed only one interview, which they consented to be audio‐recorded, and received a $25 gift voucher. Recordings were transcribed verbatim, deidentified and reviewed for detail and accuracy. Quotes include numerical coding in order of interview, with W. I. indicating RCT participants and L. C. indicating non‐RCT participants. The involvement of all AMS research officers in facilitating the trials, ensuring trust, respect, informed consent and culturally appropriate care of the patients throughout the research was essential to the success of WATCH/INFLATE and the interview study.

### 3.5 Data analysis

Transcripts were thematically analysed using a constructivist grounded theory approach, which encourages reflection on how researcher perspectives, position and privilege influence the analysis.^24^ Using NVivo 12 software (QSR), initial coding on all transcripts was undertaken by L. C., with independent co‐coding of half of the transcripts by P. A. Focused coding and early themes were determined by these authors, then reviewed and refined through discussion with the whole team, memo writing and repeated reference back to the data. L. C. led the data analysis, drawing on her insider knowledge of the community, cultural context and OM. With four years of experience working within the community screening for ear disease, L. C. had many prior discussions with families about the experience of living with OM, which aided the recognition of patterns and the creation of themes for the project. Having an Aboriginal person leading the analysis ensured that Aboriginal and Torres Strait Islander people's perspectives were privileged and interpreted through a cultural lens.^25^ Findings from the final stage of analysis were discussed with a community advisory group for the RCTs consisting of Aboriginal and Torres Strait Islander people. The input from other team members aided the translatability of the research to a wider audience.

## 4 RESULTS

**Figure 1:**
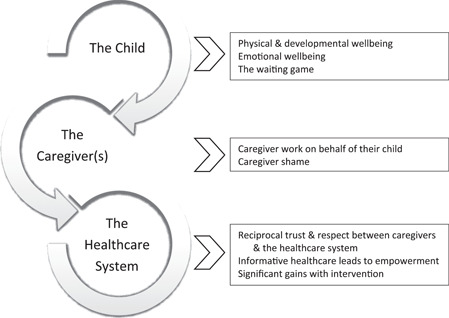
Socioemotional consequences of living with otitis media

Interviews were undertaken with 28 people, of whom 21 identified as parents (19 mothers and two fathers) and seven were carers who identified as grandparents (one grandfather, four grandmothers), a maternal aunt and a non‐related carer. Interviews ranged in duration from 12 to 53 min (26 min average). The generated themes reflect the socio‐emotional challenges and impacts on children, caregivers and interactions with the healthcare system (see Figure 1).

### 4.1 Impacts on the child's health

Caregivers described multiple impacts from OM and OM‐related healthcare on the physical, developmental and emotional well‐being of their children.

#### 4.1.1 Physical and developmental well‐being

Although concerns were raised about physical symptoms such as pain, tiredness and lethargy, more commonly, caregiver concerns focused on the consequences of OM including speech difficulties, educational challenges and developmental delays. Some caregivers noted that OM could be asymptomatic and only identified from behavioural issues or poor school performance.

I teach quite a few Indigenous students and – you can pick up a little bit on some of those that are really struggling at school… because they just don't really have the, I suppose A) the vocabulary and B) the meaning and understanding of some of the words … and so they really struggle to bring their grades up and to achieve what they could achieve if there wasn't that problem. C23LC

Some caregivers reported being uncertain and anxious about the short‐ and long‐term effects on the child's development. Families managing chronic OM often conveyed a sense of being resigned to OM being part of their life now, so caregivers focused their emotional energy on how to effectively live with the OM and its sequelae such as hearing loss in the long term.

#### 4.1.2 Emotional well‐being

It could be difficult to know if a child was being disobedient or could not hear, with caregivers and the child's teachers often making assumptions that children with OM were ‘ignorant’, ‘playing up’ or ‘joking’ about ear pain or hearing loss. This could lead to children getting distressed about being regularly in trouble for ‘not listening’ or misunderstanding what was said to them because they could not hear properly.

She's always feeling like she's in trouble, or like we're yelling at her, or she gets sad, she'll hide in the corner because she's getting yelled at …. especially the first two years, I didn't really believe her, and I was just like, ‘no, you heard me’. And then it got to a point where I worked it out and I tested her about things she likes… and she still didn't hear me about things she likes. C22LC

Two caregivers described how their children had been traumatized by their experience of OM, becoming distressed or defensive if anyone tried to touch their ears, even years later. Caregivers also explained their struggle in trying to understand a child whose speech development was affected by OM and how distressing it could be for children to be misunderstood.

With her behaviour though, she wasn't herself, but she would play up, would be a bit more than usual, and more attention, and [get] very frustrated… even to this day she still gets so frustrated with us because we don't understand her, so she cries. C25LC

#### 4.1.3 The waiting game

Persistent OM and its sequelae often required the involvement of numerous healthcare providers including allied health specialists or surgeons. Frustration was commonly expressed regarding long wait times to see these specialists and the strain that waiting placed on the whole family. It was particularly difficult in cases where hearing and speech delays complicated and delayed diagnosis or treatment of other conditions, such as Autism Spectrum Disorder. Caregivers reported that healthcare providers would sometimes delay diagnoses or treatment until ear issues had resolved, but this created additional stress and delayed outcomes for the children who had to wait for speech therapy or were deterred from attending school until after surgical treatment.

They said oh if her ear isn't, when her ears and that get done… then she can come in [to school]. Until then her behaviour is too much, and they need the [ears] fixed before she can go in yeah, a lot of mucking around, especially with the waiting, all that waiting. C22LC

### 4.2 Caregiver impacts

Caregivers described the emotional and logistical impact that having a child with OM had on them and the lengths to which they went to try to ensure that their child's needs were addressed in a timely manner.

#### 4.2.1 Caregiver work on behalf of their child

Caregivers experienced uncertainty as to how they would know when their child had OM or hearing loss and how to judge severity. They reported constantly checking and carefully watching to try to detect issues and questioned if they were misinterpreting symptoms or worrying too much or too little.

She pulls on her ear and goes, I'm sore; but she doesn't get temperatures, she doesn't get lethargic. She would just pull that a tiny bit, so it's probably a little bit sore. So, I think that something's going on that she can't hear. She says water when she pulls on it. C25LC

Feeling overwhelmed by the variability of the condition was common, but caregivers demonstrated agency finding patterns in symptoms to work out when to just watch and when to seek medical aid. They discussed their own beliefs and reasoning about whether antibiotics were indicated and which medication they preferred under particular circumstances.

It was red, but it wasn't bothering him at all then we wouldn't worry about using antibiotics. It was more about just assessing him and seeing how he feels. And if he felt fine and he was happy, then we kind of just let it go a little bit and then we'd have the antibiotic script there waiting, but we'd either not fill it or fill it, depending on how he was feeling. C16WI

When healthcare providers or the system did not meet caregiver expectations of adequate care, many caregivers described pushing back with their doctors, telling them when they disagreed with treatment decisions and requesting different treatments or quicker timeframes for surgery. One family described using a relative's address to be seen in a different medical catchment area and driving long distances to attend appointments and access surgery for OME quicker.

I would take her to the doctors and they'd just give her antibiotics or give her drops and send her home. And I kept taking her back to the doctors, same thing, day in, day out. And I got to the point where I got fed up with it. So, I took her to the hospital. C10WI

#### 4.2.2 Caregiver shame

Caregivers often said that they felt guilty when they or their family members misinterpreted OM as poor behaviour and judged their children rather than understanding the reasons for the behaviour. Some stated that they knew something was wrong, but when medical professionals dismissed their concerns, they questioned themselves and doubted their parenting ability.

I just thought that she was just a bit ignorant, you know, when I'd call out to her, she wouldn't look at me and then I'd take her to the doctor's, and she's got glue ear. I felt bad afterwards… I had to say to my mum—because mum would go, ‘She's just being ignorant’. I'd go, ‘Mum, you have to let her see your mouth move, so that she knows that you're talking to her’. C10WI

Even after the diagnosis of OM, some talked about feeling shame about seeking healthcare, concerned that their parenting would be questioned, and that having a sick child meant that they were a bad caregiver. Some described their fear of being reported to government authorities or having their children removed from their care if they went to the doctors too often with a sick child.

I just feel that everybody is watching me – it could be an easy thing for a doctor to say this child is always getting ear infections, …chest infection or something else, I need to report this [to child safety]. C3WI

### 4.3 Interacting with the health system

Caregivers had varied views of their interactions with the health system and offered insight into what they needed from the system to access appropriate care and manage their child's OM.

#### 4.3.1 Reciprocal trust and respect between caregivers and the system

The relationship with the healthcare system and providers was highlighted by caregivers as an essential component of treatment, as much as medicine or medical procedures. Caregivers spoke favourably of their interactions when they felt that they had been heard and their knowledge of the child's condition was valued by the healthcare providers in the diagnosis and OM management decisions.

Just because they can see inside their ears [the doctor thinks] that they know better… you know your child, what their pain threshold is. You'd know if your child is one of those kids that doesn't say anything until the last minute, or one of those kids that's been telling you, ‘Mum, I'm sick, mum, I'm sick, mum, I'm sick’… I think just paying a bit more respect to parents and what they bring, because if we're talking about holistic health, listening to what the parents bring to the conversation as well. C25LC

Having continuity of care from healthcare providers also helped build trust and respect in the patient–practitioner relationship. Caregivers who felt like their concerns and input was dismissed or ignored described purposely not complying with treatment, seeking other opinions or changing health services completely for all their healthcare needs.

#### 4.3.2 Informative healthcare leads to empowerment

Most caregivers described willingness to try a range of strategies to access healthcare such as seeking different opinions or investigating private pathways. However, it appeared that some had greater agency from experience or personal knowledge of how to overcome health system barriers, while others felt at a loss about how to access treatment.

Yeah, having more information really empowers you and you get more insight into what's going on and you can actually be – better manage the situation. C26LC

Communication of diagnosis and treatment through uncomplicated language empowered some caregivers to actively engage with the healthcare system. The use of diagrams to explain the ear's anatomy and process of OM was appreciated, as were discussions of treatment options and the practitioner providing their reasons for a particular recommendation. Caregivers said that they valued learning about OM and shared this knowledge with other family and community members.

You can take what you learn … and obviously go home and help your relatives as well – your cousins, nieces and nephews when they have an ear issue; you have a better understanding of what's going on, and instead of them suffering with it, you can let them know to get to a doctor and make sure. C16WI

Frustration and confusion were described by caregivers who felt that healthcare providers communicated using inaccessible medical jargon or did not explain what was happening at all. Lack of understanding about the disease and treatment steps contributed to extra emotional strain for caregivers trying to navigate through the health system on top of the day‐to‐day stresses of managing a child with OM.

#### 4.3.3 Significant gains with intervention

Despite long wait‐times for some treatments like surgery, caregivers reported feeling more comfortable when they felt that their child was being monitored closely so they could focus on managing the child's symptoms and not managing the health system. Caregivers often reported immediate improvements after surgery for OME, like children responding to sounds when returning from hospital or speaking for the first time. While speech and language consequences often took years to rectify, caregivers expressed gratitude at receiving the intervention, particularly when services responded quickly.

But she's not as bad now, she focuses a lot more. Ever since we've been getting on top of everything, including her speech therapy, she's just getting so good at like everything. C28LC

I heard stories about people saying oh yeah, you know, that their kids just started to talk when they got [ear surgery] … it really was, it was in 1‐2 weeks. He started to talk. C21LC

## 5 DISCUSSION

The research findings add to the very limited previous research of First Nations people living with OM, providing further insight into the daily experiences and key concerns of caregivers within this population. For many Aboriginal and Torres Strait Islander families, OM was not experienced as an isolated episode, but a fluctuating chronic condition, which could become a complicated journey of watching, waiting, treating and repeating. Caregivers shared a wide variety of concerns and factors that they felt were important in managing their child's OM and navigating the health system.

Although it is important to assess symptoms like pain or hearing loss, when many children present with minimal symptoms, it is apparent that a greater focus is needed on the emotional, behavioural and functional impacts of OM.^26^ The holistic perspective of health in Aboriginal and Torres Strait Islander culture that considers the interconnection and relatedness of all things (physical, social, psychological, spiritual and environmental) is a valuable mind set for considering OM management and treatment.^14^ Two studies of caregivers of First Nations children in Greenland^10^ and in Western Australia^6^ reflected on their experiences of coping with CSOM, and the emotional impacts on their children of being embarrassed due to the smell and sight of ear discharge and being bullied by other children because of their CSOM. Comparatively, our findings show that even in the absence of discharge, children with OM can be treated negatively by others when OM‐impaired speech is misunderstood, and behaviour caused by OM‐related hearing impairment is characterized (and often punished) as intentional bad behaviour.

OM is an emotionally charged experience for the children suffering from the disease and also for their caregivers. Research focusing on the caregiver has frequently exposed the strain of the financial and logistical impacts of OM such as time for attending appointments, collecting and administering medication, absences from work and disrupted sleep.^7^, ^11^, ^27^ However, qualitative studies have also highlighted that the presence and continued reoccurrence of OM provoke feelings of guilt, helplessness, despair and a sense that they or the healthcare system are failing the child.^8^, ^10^ Self‐blame can be focused on the belief that the caregiver actively caused the OM through environmental and family factors,^10^ or shame can centre on the delayed diagnosis and misinterpretation of the child's behaviour rather than OM aetiology.^8^ Our findings show that these experiences of shame and self‐blame commonly occur in Aboriginal and Torres Strait Islander families and that these strong negative emotions are often influenced by outside sources.

In First Nations communities, the sociocultural impact of having a child with OM plays a big part in the experiences of shame for these caregivers.^1^, ^6^, ^10^ Government policies of child removal and experiences of racism were cited by caregivers as affecting their self‐worth, confidence in their parenting and their unwillingness to seek medical help for fear of having their children removed from their care. These prevailing fears and expectations of judgement, coupled with racially motivated poor care by health professionals, are not unique to OM. Carers of Aboriginal children with disabilities reported similar hesitancies to access care when providers were perceived to be judgemental or disrespectful towards their family or other Aboriginal families.^28^

Caregivers' experience of the healthcare system is often fleeting, brief and paternalistic.^29^ Access and institutional bias still remain a major barrier to care, and the current findings provide insight concerning steps to reduce this barrier. Caregivers are not experts on OM when their child first develops the problem, but our findings indicate that they have a strong desire to learn about OM and be involved in the ear and hearing health pathway. Caregivers managing persistent OM demonstrated perseverance in knowledge‐seeking to identify specific symptoms in their child and take on an active role in treatment decisions. Canadian First Nations caregivers expressed similar views about adopting an active approach to understanding the meaning of behaviours and using this knowledge to engage with the healthcare system more confidently for the benefit of the child.^30^ Developing this expertize, agency and self‐confidence in the management of OM requires the support of the health system and healthcare providers.^8^ Caregivers in our study described seeking healthcare providers' support to effectively self‐manage the condition and its consequences once out of the clinic and at home, a common desire for First Nations communities irrespective of medical conditions and patient ages.^31^ Such support requires open and generous communication from healthcare providers, using clear language about the disease and its management.^28^, ^31^ The respect that is felt from the interaction, including validation from a clinician, strengthens relationships with Aboriginal and Torres Strait Islander patients, leading to reciprocal respect in the form of treatment adherence and return consultations.^8^, ^28^, ^32^, ^33^

### 5.1 Strengths and limitations

The strengths of this study include the fact that it was community driven, inspired by conversations with caregivers during ear and hearing screening, engaged Aboriginal and Torres Strait Islander health staff and expertize and also involved analysis led by an Aboriginal researcher^25^ living in a community participating in the WATCH/INFLATE trials. The trust that was pre‐established between the AMS research officers and the community before interviewing provided a culturally safe relationship where participants could comfortably accept or decline participation and likely increased participation as well as the level of sharing and honesty. The study has contributed to the health research reform agenda of privileging Indigenous voices by investigating the experience of OM for caregivers and Aboriginal and Torres Strait Islander children through the leadership and ownership of the project by an Aboriginal researcher.^14^ As lead author, L. C. utilized her cultural, personal and professional knowledge to examine OM stories shared by community to create new knowledge that increased the participation and control of the research agenda by the Aboriginal and Torres Strait Islander community.^25^ The research also maintained the notion of Rigney's political integrity,^16^ with the researchers ensuring that their roles as clinical educators, clinicians and health liaison were fulfilled during and after the research interview. The research therefore has had immediate effects on the Aboriginal and Torres Strait Islander communities involved in the research.

This study has several limitations. It was beyond the scope of this study to include interviews with children and this may be a potential inclusion in future research. Participants were limited to patients already engaging with the AMS and with the AMS research officers. This was also a strength in terms of the trust that had been established with participants before the interviews. Participants often stated that they consented because of their desire to help the AMS research officer and give back to the AMS and community by sharing their experiences. Some interviews were conducted by nonindigenous interviewers, which could have impacted on what and how much was shared in the interview depending on the participant's preference for cultural connection or anonymity. The different styles of interviewing and personal frames for interpreting responses between the multiple interviewers may have also affected the content. COVID‐19 pandemic restrictions on patient contact encouraging telehealth and limits on in‐person attendance at clinics affected our ability to conduct interviews in person during lockdown periods, requiring interviews to be conducted predominantly over the phone.

## 6 CONCLUSION

Interviews of the caregivers of Aboriginal and Torres Strait Islander children with OM has revealed some similarities of experiences with other caregivers but also highlighted the particular experiences of this group. Wide‐ranging effects are documented in this study that go beyond the common symptoms of OM to include important but often overlooked socioemotional impacts and the complexities of navigating the healthcare system as well as the disease itself. Agency and care are evident among caregivers of Indigenous children with OM, demonstrating the centrality of confidence in managing OM to cope in the longer term. This, however, is still dependent upon a healthcare system and healthcare providers that are capable of promoting respectful, supportive and well‐communicated healthcare for Aboriginal and Torres Strait Islander people. This qualitative examination of caregiver's experience makes a vital contribution towards understanding the personal, political and social context of ill health and healthcare provision.

## CONFLICTS OF INTEREST

The authors declare that there are no conflicts of interest.

## AUTHOR CONTRIBUTIONS

**Letitia Campbell**: conceptualization (lead), data curation (lead), formal analysis (lead), funding acquisition (equal), investigation (equal), methodology (supporting), project administration (equal), resources (equal), writing – original draft (lead), writing – review and editing (lead). **Jennifer Reath**: conceptualization (supporting), funding acquisition (equal), methodology (equal), project administration (equal), supervision (supporting), writing – review & editing (supporting). **Wendy Hu**: conceptualization (supporting), funding acquisition (equal), investigation (equal), methodology (equal), project administration (equal), supervision (supporting), writing – review and editing (supporting). Hasantha Gunasekera: conceptualization (supporting), funding acquisition (equal), methodology (equal), project administration (equal), writing – review and editing (supporting). **Deborah Askew**: conceptualization (supporting), funding acquisition (equal), methodology (equal), project administration (equal), writing – review and editing (supporting). **Chelsea Watego**: conceptualization (supporting), funding acquisition (equal), investigation (equal), methodology (equal), resources (equal), supervision (supporting). **Kelvin Kong**: conceptualization (supporting), funding acquisition (equal), methodology (equal), supervision (supporting), writing – review and editing (supporting). **Robyn Walsh**: conceptualization (supporting), funding acquisition (supporting), investigation (equal), methodology (equal), project administration (equal), writing – review and editing (supporting). **Kerrie Doyle**: supervision (supporting), writing – review and editing (supporting). **Amanda Leach**: conceptualization (supporting), funding acquisition (supporting), writing – review and editing (supporting). **Claudette Tyson**: resources (equal), supervision (supporting), writing – review and editing (supporting). **Penelope Abbott**: conceptualization (supporting), data curation (supporting), formal analysis (supporting), funding acquisition (equal), investigation (equal), methodology (equal), project administration (equal), supervision (lead), writing – original draft (supporting), writing – review and editing (supporting).

## Data Availability

The data that support these findings are not shared for cultural reasons.
